# Microstate D as a Biomarker in Schizophrenia: Insights from Brain State Transitions

**DOI:** 10.3390/brainsci14100985

**Published:** 2024-09-28

**Authors:** Rong Yao, Meirong Song, Langhua Shi, Yan Pei, Haifang Li, Shuping Tan, Bin Wang

**Affiliations:** 1 College of Computer Science and Technology (College of Data Science), Taiyuan University of Technology, Taiyuan 030024, China; yaorong@tyut.edu.cn (R.Y.); 2024319018@link.tyut.edu.cn (M.S.); 2023510396@link.tyut.edu.cn (L.S.); peiyan0433@link.tyut.edu.cn (Y.P.); lihaifang@tyut.edu.cn (H.L.); 2 Psychiatry Research Center, Beijing Huilongguan Hospital, Peking University Huilongguan Clinical Medical School, Beijing 100096, China; shupingtan@126.com

**Keywords:** microstate, brain network, synchronization, controllability, pinning nodes, schizophrenia

## Abstract

**Objectives.** There is a significant correlation between EEG microstate and the neurophysiological basis of mental illness, brain state, and cognitive function. Given that the unclear relationship between network dynamics and different microstates, this paper utilized microstate, brain network, and control theories to understand the microstate characteristics of short-term memory task, aiming to mechanistically explain the most influential microstates and brain regions driving the abnormal changes in brain state transitions in patients with schizophrenia. **Methods.** We identified each microstate and analyzed the microstate abnormalities in schizophrenia patients during short-term memory tasks. Subsequently, the network dynamics underlying the primary microstates were studied to reveal the relationships between network dynamics and microstates. Finally, using control theory, we confirmed that the abnormal changes in brain state transitions in schizophrenia patients are driven by specific microstates and brain regions. **Results.** The frontal-occipital lobes activity of microstate D decreased significantly, but the left frontal lobe of microstate B increased significantly in schizophrenia, when the brain was moving toward the easy-to-reach states. However, the frontal-occipital lobes activity of microstate D decreased significantly in schizophrenia, when the brain was moving toward the hard-to-reach states. Microstate D showed that the right-frontal activity had a higher priority than the left-frontal, but microstate B showed that the left-frontal priority decreased significantly in schizophrenia, when changes occur in the synchronization state of the brain. **Conclusions.** In conclusion, microstate D may be a biomarker candidate of brain abnormal activity during the states transitions in schizophrenia, and microstate B may represent a compensatory mechanism that maintains brain function and exchanges information with other brain regions. Microstate and brain network provide complementary perspectives on the neurodynamics, offering potential insights into brain function in health and disease.

## 1. Introduction

Schizophrenia is a brain disorder with an incompletely elucidated etiology which is characterized by a high prevalence, a high relapse rate and a high disability rate [1]. The early stages often cause an impairment in brain function, resulting in behavioral, cognitive, thinking and emotional dysregulation. During the progression and persistence of the pathological process, irreversible structural damage can be caused to the brain [2]. The core feature of schizophrenia is cognitive difficulty [3], and short-term memory issues are one of the key pathogenic mechanisms in schizophrenia. Hence, in view of the compared differences between patients with schizophrenia and healthy controls in short-term memory tasks, they are of great significance for understanding the pathology of schizophrenia and improving clinical intervention.

The brain exhibits multiple dynamic patterns during healthy functioning and mental illness. The psychotic symptoms that accompany schizophrenia are widely recognized as being closely related to dysfunctional interactions between brain regions [1]. Several studies assessed the brain network changes during a cognitive task in people with schizophrenia and healthy controls. For example, Gomez et al. [4] found alterations in the structural and functional networks in schizophrenia patients and a lack of significant relationships between these alterations, suggesting that different types of network abnormalities exist in different groups of schizophrenia patients. Zhao et al. [5] found that theta band power, as well as brain network topology attribute values, were significantly lower in schizophrenic patients than in healthy controls. Yang et al. [6] found that compared with healthy controls, schizophrenia patients showed a higher state of weak sparse connectivity, which was positively correlated with negative symptoms. Despite the advances made so far, the ways the underlying network structure can support, shape, and constrain various dynamic patterns in different brain states are still unknown and is a central challenge in network neuroscience [7].

The EEG microstate, as a significant representation of the brain’s physiological activities, reflects the potential topography of the scalp EEG in an organized manner, and the transitions between different states directly reflect the dynamic changes in brain activities. EEG microstates can be classified into four typical types, A, B, C, and D. The four microstates are categorized as auditory network (microstate A), visual network (microstate B), saliency network (microstate C), and frontal-parietal network (microstate D) in the synchronized EEG-fMRI literature [8,9].

Due to their high stability and reliability [10], microstates have been widely used to explore brain dysfunction in various neurological conditions, such as bipolar disorder (BD), schizophrenia (SZ), depression (DP), and Alzheimer’s disease (AD). In related studies on schizophrenia, Cruz et al. [11] found that schizophrenic patients and their siblings had an increase in microstate class C and a decrease in microstate class D compared to heathy controls. Microstate D is associated with working memory and cognitive control, and its reduction indicates a dysregulation of executive functioning in patients. The main manifestation is a lack of control of core cognitive functions, which leads to the appearance of typical psychotic symptoms. Notably, this pattern of microstate change did not manifest significant differences between patients with first-episode schizophrenia and patients with long-term schizophrenia. Seitzman et al. [12] explored changes in the temporal characteristics of four microstates through behavioral manipulation. They found a significant increase in microstate B when participants transitioned from eyes closed to eyes open, confirming the hypothesis that microstate B is associated with the visual system. Sun et al. [13] explored the dynamics of EEG microstates in first-treatment first-episode schizophrenia. They found that, compared to the HC group, the duration, occurrence, and contribution of microstate C were increased in the SZ group. In contrast, microstate D was decreased, which may indicate impaired functioning of the frontoparietal and dorsal attention networks in schizophrenia. These results provide new evidence that the dynamic abnormalities of the EEG microstate play an important role in the in-depth understanding of the pathological mechanisms of schizophrenia.

The brain network and microstate provide complementary perspectives on the neurodynamics underlying cognitive tasks in schizophrenia. However, the relationship between network dynamics and different microstates has not yet been investigated during short-term memory tasks. In this paper, we applied microstate, brain network and control theories in order to understand the microstate characteristics of short-term memory tasks, aiming to reveal the network dynamics behind each microstate, and explored the types of microstate and brain regions that have the most influence on abnormal changes in state transitions in the schizophrenic brain. First, we identified each microstate and analyzed the microstate abnormalities in schizophrenia during short-term memory tasks. Next, we investigated the network dynamics underlying each of the four microstates, and found a previously unknown relationship between network dynamics and microstate. Finally, we utilized control theories to confirm that abnormal changes in brain state transitions were driven by specific microstate and brain regions in schizophrenia. These will offer new insights into differential microstate mechanisms of cognitive control between SZ and HC groups, and provide a theoretical foundation for the application of microstates to network intervention responses in the future.

## 2. Materials and Methods

### 2.1. Overview

**Figure 1:**
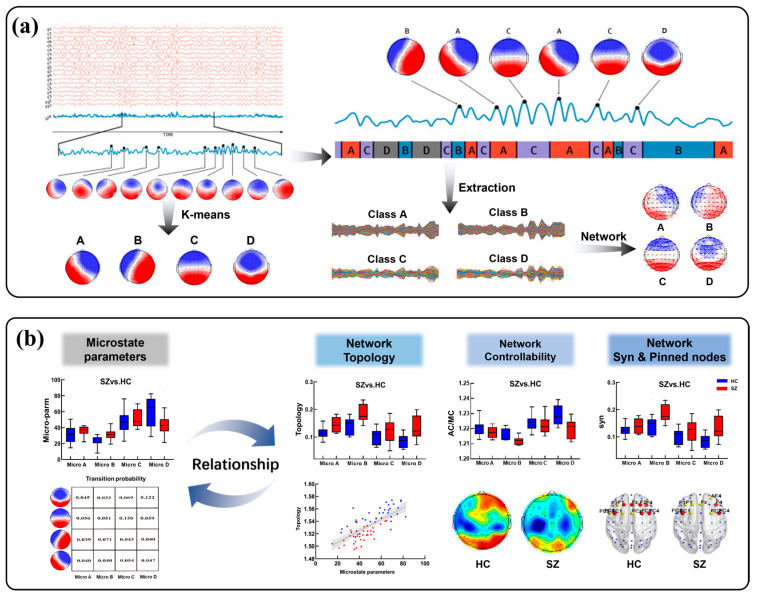
Method flowchart. (**a**) Construction of the microstate network model. (**b**) Analysis of the relationship between microstates and network dynamics.

As shown in Figure 1, the overall method flowchart contains two main components. (a) The construction of microstates network model. This is mainly divided into three steps: EEG data preprocessing, microstate time series extraction and network construction. Specifically, we used the GFP peaks to generate the topography of electrode arrays, and the topography was grouped into clusters by a k-means clustering algorithm to obtain four microstates (A, B, C, and D). Then, the corresponding time series were extracted and the brain network was constructed according to Algorithm 1. (b) The analysis of the relationship between microstates and network dynamics. Firstly, the microstate parameters were calculated for HC and SZ participants, respectively (Supplementary Materials). Secondly, the potential relationship between microstates and network dynamics was explored from the perspectives of network topology, network controllability, network synchronization and pinning control, respectively.
**Algorithm 1** Microstate Network ModelStep 1: According to the microstate template, the microstates A, B, C, and D time series are extracted for each participant.
Step 2: The x and y channels’ signals are transformed by the Hilbert transformation:$\tilde{x}\left(t\right)=\frac{1}{\pi t}*x\left(t\right)=\frac{1}{\pi }\int_{+\infty }^{-\infty }\frac{x(\tau )}{t-\tau }d\tau$$\tilde{y}\left(t\right)=\frac{1}{\pi t}*y\left(t\right)=\frac{1}{\pi }\int_{+\infty }^{-\infty }\frac{y(\tau )}{t-\tau }d\tau$Step 3: The phase of the x and y channels’ signals are calculated from the original signals ($x\left(\tau \right),y(\tau )$) and transformed into conjugate signals ($\tilde{x}\left(t\right),\tilde{y}\left(t\right)$):$\varphi_{x}\left(t\right)=arctan\frac{\tilde{x}\left(t\right)}{x\left(t\right)}$$\varphi_{y}\left(t\right)=arctan\frac{\tilde{y}\left(t\right)}{y\left(t\right)}$Step 4: According to the phases of the two signals, the average phase difference between the x and y signals is ${PLV}_{xy}=\frac{1}{Len}\left|\sum_{n=1}^{N}exp(j(∆\varphi_{n}(t)))\right|$where $∆\varphi_{n}=\varphi_{x}(t)-\varphi_{y}(t)$ is the phase difference between *x* and *y* channels at time *t*, and Len represents the length of the time series, PLV∈[0,1].

### 2.2. Participants and Processing

A total of seventy participants were recruited for this study, including thirty-five patients who had just received their first diagnosis of schizophrenia. The study groups did not differ significantly in age, gender, or education level. They all had normal levels of visual acuity (including corrected vision), no abnormalities in color vision, and were right-handed. In addition, none of the participants had a history of drug abuse or any other neuropsychiatric disorders prior to the study. The memory-scanning task (SMTS) employed in this study was derived from the modified Sternberg paradigm [14,15]. The experiment was conducted in accordance with the ethical standards laid down in the 1964 Declaration of Helsinki, and the protocol was approved by the ethics committee of Beijing Hui Long Guan Hospital (HLG[2015]103, dated 10 June 2015).

In this study, a NeuroScan 64-lead EEG system was used to collect EEG data, with a sampling frequency of 500 Hz. Initially, the data were re-referenced using the average reference transformation, and obvious artifacts were removed by visual observation. Then, a finite FIR filter was used to band-pass-filter the data, with a high-pass cutoff frequency of 0.5 Hz, a low-pass cutoff frequency of 60 Hz, and a notch filter of 49–51 Hz to eliminate power frequency interference. Furthermore, the eye movements, eyeblinks, and muscle artifacts in the data were removed using the ICA algorithm. And we used the surface Laplace transform method (MATLAB (2014bMathWorks, Natick, MA, USA) toolbox CSD) [16] to remove the mixing effect of volume conduction. Importantly, we focused on the theta (4–7 Hz) frequency band, because it is closely related to several key domains, including microstate [17], top-down cognitive control [18], memory processes [19], neural synchronization [20], and topological connectivity patterns in brain networks [21].

### 2.3. Microstate Topographic Maps

We performed a microstate extraction following a protocol employed in previous studies [11]. Specifically, the global field power (GFP) of the 60 electrodes was calculated, and the local maximum values of the GFP were obtained. These, respectively, represented the moments of strongest field strength and highest topographical signal-to-noise ratio. The GFP is the root of the mean of the squared potential differences at all electrodes from the mean of instantaneous potentials across electrodes, and can be calculated as

(1) GFP=(∑iK(Vi(t))−Vmean(t)2)N

where $V_{i}\left(t\right)$ indicates the instantaneous potentials at electrode i at the given time t, $V_{mean}(t)$ reflects the average instantaneous potentials across all electrodes at the same time t, and N denotes the total number of electrodes involved.

**Figure 2:**
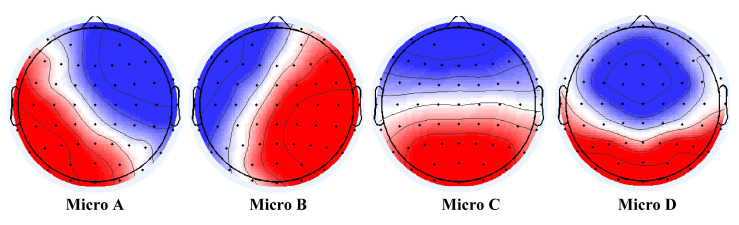
Microstate topographic maps. Red indicates positive values and blue indicates negative values (can be inverted).

The peaks of the GFP are employed to generate topographic maps of the electrode array, and the topographic maps are grouped into clusters. The most commonly reported microstate clustering methods are terrain atomisation-aggregation hierarchical clustering (TAAHC) [22] and k-means clustering [23,24]. Khanna and colleagues [25] demonstrated the consistency of these clustering methods. We applied the k-means clustering algorithm to the set of combined samples from the schizophrenia patients and healthy controls, to obtain four microstate topographic maps. The polarity of the EEG microstate topographic maps was ignored. The four microstate topographic maps are exhibited in Figure 2, and are labeled (A–D) in accordance with the previous literature [11]. Microstate A involved right-frontal to left-posterior activity, microstate B involved left-frontal to right-posterior activity, microstate C involved frontal to occipital activity, and microstate D involved mostly frontal and medial activity to slightly less occipital activity compared to class C [26].

### 2.4. Microstate Network Model

The nodes in the microstate network comprised 60 electrode channels, while the edges represented the phase-locking values (PLV) calculated between all channels. Because EEG data can be affected by transient amplitude changes such as eye movements, the PLV is considered more suitable for analyzing functional coupling between EEG signals [27]. The Microstate Network Model was calculated using Algorithm 1, as follows. We calculated microstate network topologies (including local efficiency (Eloc), global efficiency (Eg), clustering coefficient (Cp), characteristic path length (Lp), and small-worldness (Sw)) to examine both the global and local characteristic variations.

### 2.5. Network Local Controllability

Controllability [28] assesses the most influential set of brain regions involved in promoting the brain state transitions, including Average Controllability (AC) and Modal Controllability (MC), each with different brain states. The AC quantifies how easy it is for a single node to drive the brain into an easy-to-reach state, such as inducing a relaxed state with minimal energy. The higher AC indicates that this region is capable of driving the brain to easy-to-reach states with lower energy. Conversely, the MC quantifies how easy it is for a single node to drive the brain into hard-to-reach states, such as transitioning from sleep to memory states, which require a higher amount of energy. The higher MC indicates that this region is capable of driving the brain to hard-to-reach states with a lower amount of energy.

The measurement of AC is based on the trace of the controllability Gramian, Trace ($W_{k}$), as follows:

(2) $$W_{k}=\int_{0}^{\infty }e^{At}B_{k}B_{k}^{T}e^{A^{T}t}dt$$

where A is the microstate network matrix and $B_{\kappa }$ is the control input matrix; we select a single node κ at a time, and thus $B_{\kappa }$ is simplified as a one-dimensional vector such as $B_{\kappa }=(1,0,0,\ldots {)}^{T}$. By calculating the trace of the controllability Gramian, the responsiveness of the whole brain to control inputs can be evaluated.

The measurement of MC is based on the eigenvalues and eigenvectors of the microstate network matrix, as follows:

(3) $$\phi_{i}=\sum_{j=1}^{N}\left(1-\lambda_{j}^{2}\left(A\right)\right)v_{ij}^{2}$$

where $\lambda_{j}$ is the jth eigenvalue of matrix A, and $v_{ij}$ is an element of the eigenvector matrix of A. MC emphasizes the brain’s response characteristics under different modes, reflecting the importance of nodes in controlling the overall behavior of the brain.

### 2.6. Network Synchronization

The synchronization of neural activity in a healthy brain is one of the key mechanisms which ensures proper brain function. It integrates and coordinates neural activity on both temporal and spatial scales [29], enabling the brain to efficiently process information and to perform complex activities, including cognition [30], perception, and movement [31]. Abnormalities in synchronization activity, such as too strong, too weak, or improperly organized synchronization, may disrupt healthy brain function and lead to a range of neurological disorders or cognitive issues.

The most widely used and most effective synchronization evaluation metric is the ratio of the second-smallest eigenvalue to the maximum eigenvalue of the network in the Laplacian matrix [29]. This metric was also used in the current research. A Laplacian matrix is defined as L=D−A, where D is the diagonal matrix and $d_{ii}$ the ith node’s degree. The eigenvalues and eigenvectors are obtained by applying eigen-decomposition to the Laplacian matrix L of the microstate brain network. Based on the nature of the Laplacian matrix of the network, we can obtain the eigenvalue relationship $\lambda_{max}>\ldots >\lambda_{2}>\lambda_{1}>0$, and they are all real numbers. We were particularly interested in the eigenvalue ratio M=λ2(L)λmax⁡(L), a metric that helps characterize the synchronization dynamics of the network, where $\lambda_{2}$ is the second-smallest eigenvalue and $\lambda_{max}$ is the maximum eigenvalue. In general, a brain network with a greater M is more synchronized; that is, its nodes’ states are more likely to be synchronized and vice versa.

### 2.7. Network Pinned Nodes

Pinning control [32] represents a feedback strategy based on network coupling, aimed at propagating stabilizing effects through interactions among nodes to regulate the dynamic behavior of the whole network. In neuroscience, synchronization pinning control refers to guiding the network to the synchronization state by selectively regulating or manipulating key nodes [33]. Specifically, the synchronization state is altered by applying control energy to specific nodes so that the state changes of these nodes can be propagated to other nodes through network coupling. For instance, when performing a certain cognitive task, all relevant brain regions involved in the task are activated for efficient dynamic interaction, and their cooperative effect is promoted to enhance the information processing ability and response speed, thereby improving the execution efficiency of the cognitive task [34].

This raises the question of which regions within the brain network play the most important role in altering the state of brain synchronization. Answering these questions requires knowledge about (i) the brain connectivity matrix and (ii) the dynamic interactions between brain regions, that is, their dynamics. Mathematically, we can identify the key nodes that change the synchronization state given the brain connectivity matrix and its dynamical equations, which are referred to as synchronization pinned nodes. The microstate pinned nodes scheme was proposed, as follows in Algorithm 2. The algorithm characterizes the dynamic interactions of the nodes (network A), establishes the relationship between the feedback gain and the network topology by controlling intensity $c_{s}$, and optimizes gain value matrix K by linear matrix inequality (LMI) to select the nodes that are most influential in changing the synchronization state. For the interested reader, a previous work [15] provided a few mathematical intuitions that might facilitate a deeper understanding of the presented concepts.
**Algorithm 2** Network Pinned Nodes SchemeInput: microstate matrix A, control strength $c_{s}$, pinned nodes set $p_{s}=\emptyset$;Step 1: Define parameters a, b, ε>0, and calculate network node importance matrix P, Q;Step 2: Using the linear matrix inequality (LMIs), calculate the optimal feedback gain matrix K such that the maximum eigenvalue $\lambda_{max}$ of the matrix R achieves the minimum value:$K_{optimal}=\left\{K|\min\left(\lambda_{max}\left(R\right)\right),R=A-K\right\}$$LMIs=\left\{\begin{array}{c} 0<K\\ K<c_{s}\\ c_{s}-\varepsilon <D^{T}KD\\ D^{T}KD<c_{s}+\varepsilon \\ A-K<\lambda_{min}I \end{array}\right.$$D={\left[{p_{1}}^{a}{q_{1}}^{b},{p_{2}}^{a}{q_{2}}^{b}\ldots \ldots {p_{N}}^{a}{q_{N}}^{b}\right]}^{T},\varepsilon >0$$K=diag\left\{k_{1},k_{2},\ldots ,k_{N}\right\}$where $k_{i}$ is the feedback gains of node i, and $k_{i}$ > 0, $p_{i}$, $q_{i}$ is the importance of node i, and $a,b\in \left[-1,0\right]$.Step 3: Sort the nodes by descending order of gains from K,and select the top 10 nodes with the highest gain values $k_{i}$ and add them to $p_{s}$.

### 2.8. Statistical Analyses

An independent samples *t*-test was used to assess the significant differences between SZ and HC groups on parameters such as microstate features, topology, controllability, synchronization and pinned nodes. A *p*-value less than 0.05 was considered statistically significant. Pearson correlation was used to assess the relationship between the microstate topographic maps and network dynamics.

## 3. Results

### 3.1. Analysis of Microstate Network Topology

**Figure 3:**
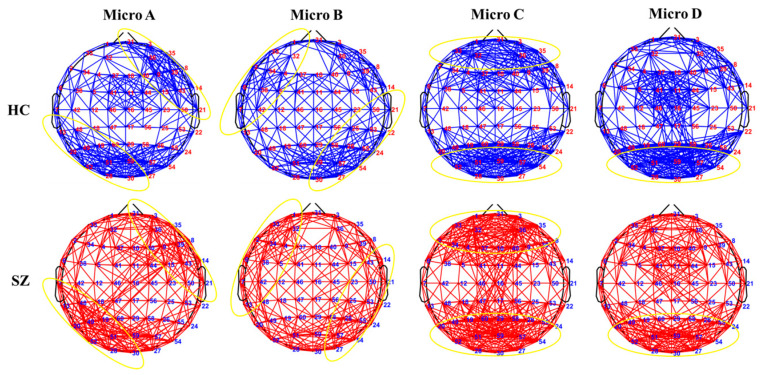
Average microstate connectivity in SZ and HC groups. The Blue color is HC group, the red color is SZ group, yellow circles indicate consistency with the microstate distribution.

**Figure 4:**
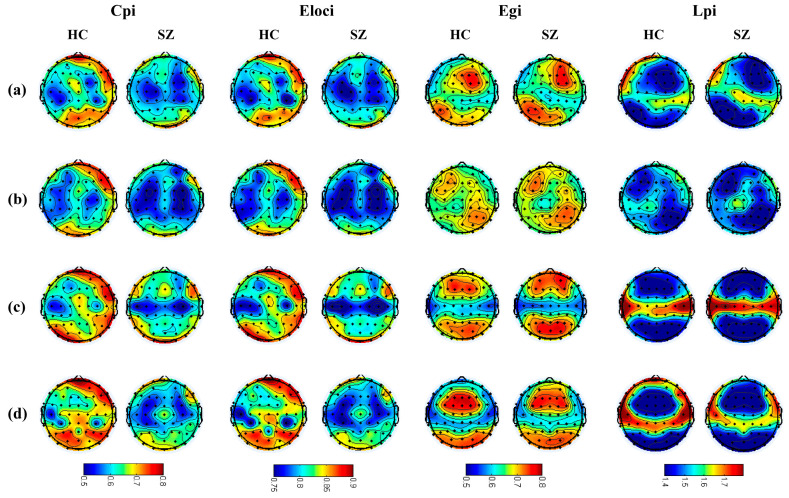
Topographic maps of the four microstate topologies. Figures (**a**–**d**) in this Figure represent microstates A, B, C, and D, respectively.

**Figure 5:**
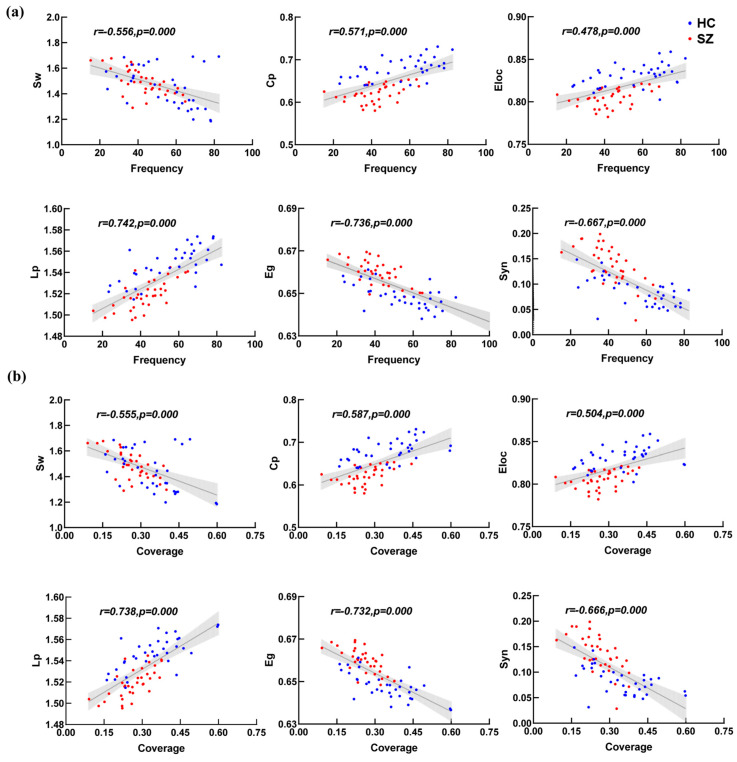
Correlation analysis of topology and parameters of microstate D. Figures (**a**,**b**) in this Figure represent frequency and coverage, respectively. Blue dots are healthy participants, red dots are schizophrenia.

We compared the average microstate connectivity between the HC and SZ groups, as shown in Figure 3. To facilitate the visualization of the networks, a threshold was applied: only those connections with a PLV higher than 0.55 were depicted. The results show that the distribution of each microstate’s connectivity was consistent with the corresponding microstate topographic map. To further explore whether the microstate topology distribution conforms to the microstate topographic map distribution, the topology topographic maps for each microstate of the SZ and HC group are shown in Figure 4. We found that the distributions of the topographic maps for Lp and Eg were consistent with the corresponding microstate topographic maps and that the differences were mainly reflected in the values. Surprisingly, it is evident that the distributions of the topographic maps of Cp and Eloc did not obey the corresponding microstate topographic maps, and their difference was only significant in the right prefrontal and parietal-occipital lobes. The Pearson correlations between the microstate topologies and their basic parameters were calculated, respectively, as demonstrated in Figure 5, and it is revealed that there was a significant correlation between the microstate D and the microstate topologies.

### 3.2. Analysis of Microstate Network Controllability

**Figure 6:**
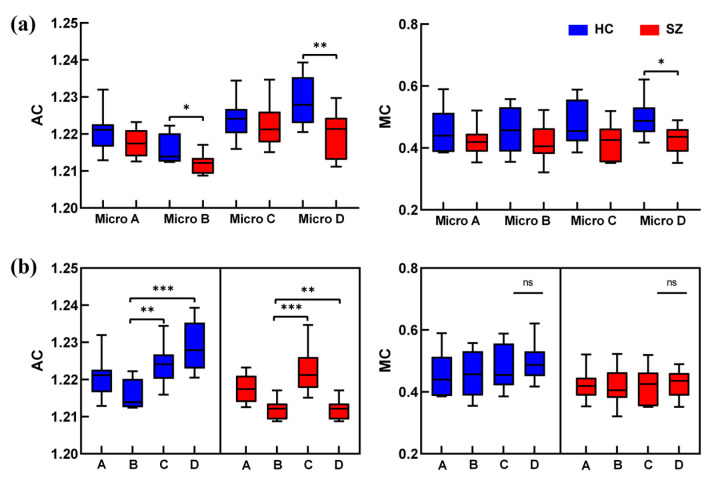
AC and MC analyses of microstates. Figures (**a**,**b**) represent AC and MC analyses of microstates intragroup and intergroup, respectively. The red color is for the SZ group and the blue color is for the HC group. Significant differences under all conditions * *p* < 0.05, ** *p* < 0.01, *** *p* < 0.001.

The intergroup and intragroup effects of the controllability of the whole brain for the four types of microstates were counted separately, as illustrated in Figure 6. The results showed that the AC of microstate B and microstate D was significantly decreased, and that the MC of microstate D was significantly decreased in the SZ group. Significant intra-group differences in the AC were observed mainly in microstates B, C, and D, and the descending order of the AC of microstates was D > C > B in HC group, whereas the descending order of microstates was C > D > B in the SZ group. And there was no significant difference within the MC group (*p* > 0.05).

**Figure 7:**
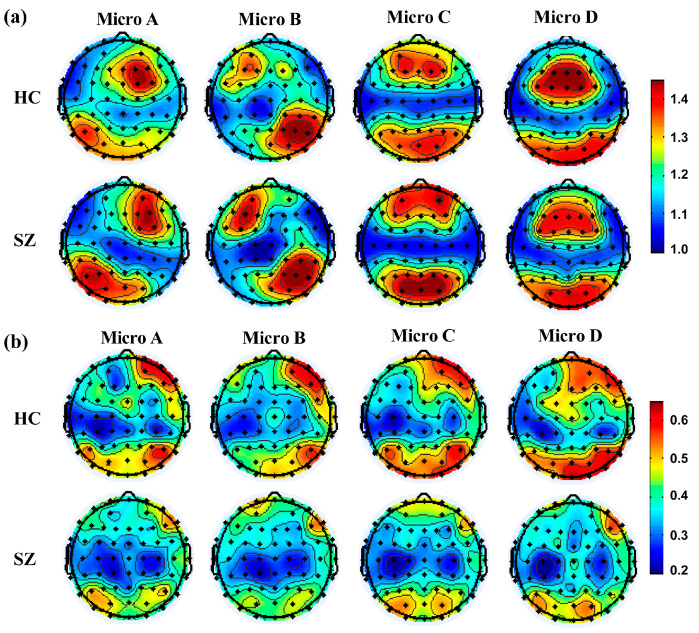
Microstate topographic map distribution. Figures (**a**,**b**) represent AC and MC, respectively.

Figure 7 shows that the distribution of nodes with high AC was consistent with the distribution of the corresponding microstate topographic maps, whereas the distribution of nodes with high MC was not consistent. Specifically, the results showed that the AC was significantly increased in the left-frontal of microstate B (4/F3, 5/FC3, 32/AF3, 37/F1) and significant decreased in the frontal (10/Fz, 37/F1, 40/F2) and occipital lobes (20/P7, 27/O2, 30/Oz, 54/PO8) of microstate D in the SZ group. However, the MC was significantly lower in the right-frontal (3/Fp2, 10/Fz, 31/Fpz, 35/AF8, 36/AF4, 40/F2) and parieto-occipital lobes (20/P7, 26/P4, 27/O2, 30/Oz, 51/PO3, 52/PO7, 54/PO8, 55/P6, 57/PO4, 59/POz) in the SZ group. For microstate D, high AC nodes were concentrated in the medial frontal and parieto-occipital lobes, and high MC nodes were concentrated in the right-frontal and occipital lobes.

### 3.3. Analysis of Microstate Network Synchronization

**Figure 8:**
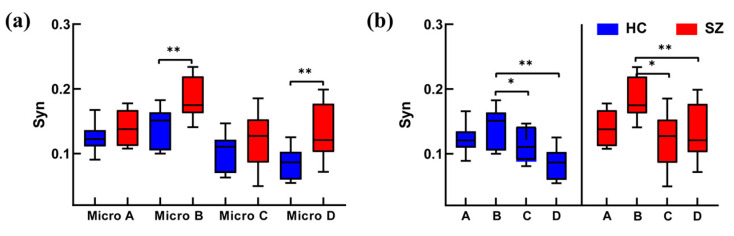
Synchronization analysis of microstates. Figures (**a**,**b**) represent synchronization analysis of microstates in the intergroup and intragroup, respectively. The red color is for the SZ group and the blue color is for the HC group. Significant differences under all conditions * *p* < 0.05, ** *p* < 0.01.

The intergroup and intragroup effects of the microstate synchronization were counted separately, as shown in Figure 8. The results showed that the Syn of the SZ group was greater than that of HC group. Specifically, the Syn of microstate B was stronger than that of other three microstates, except that microstate D was weakest in the HC group (B > A > C > D). However, the Syn of microstate B was stronger than that of other three microstates, except that microstate C was weakest in SZ group (B > A > D > C). These findings suggest that the Syn of microstate D was significantly enhanced and higher than that of microstate C in the SZ group.

### 3.4. Analysis of Microstate Network Pinned Nodes

**Figure 9:**
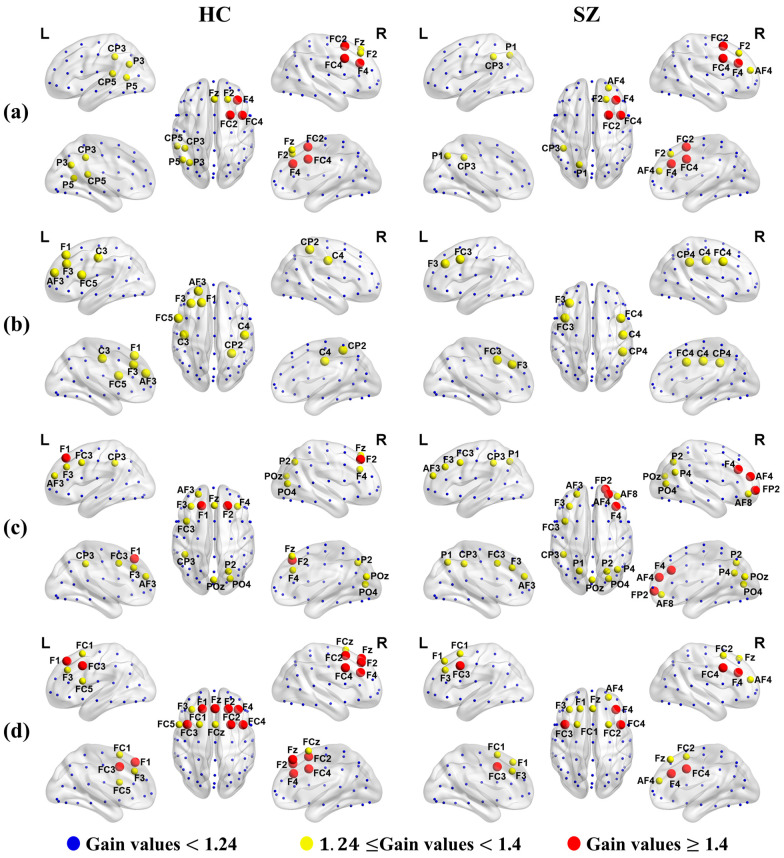
Distribution of the pinned nodes set. Figures (**a**–**d**) represent microstates A, B, C, and D, respectively. Red represents the pinned nodes belonging to the first level (node’s gain value was greater than 1.4), yellow represents the pinned nodes belonging to the second level (node’s gain value was greater than 1.24 and less than 1.4), and blue represents the pinned nodes belonging to the third level (node’s gain value was less than 1.24).

Applying Algorithm 2 to each microstate average brain network of the SZ and HC groups, we obtained the gain value for each node. Importantly, the high or low gain value of each node was positively correlated with its priority. For comparison, the pinned nodes are marked in red and yellow in Figure 9 after we set the threshold of the pinned nodes’ gain values to 1.24. The results showed that the distribution of the pinned nodes for the average of all the individual networks in each microstate was consistent with the corresponding microstate topographic map.

Furthermore, the priorities of the pinned nodes of microstates B and D were significantly different between the SZ and HC groups, but there were no significant differences in microstates A and C. Specifically, the pinned nodes of microstate B in both SZ and HC groups were distributed along a left-frontal to right-posterior direction, which is consistent with the distribution in the microstate B topographic map. Notably, the priority of the left-frontal region was higher than that of the right-posterior in the HC group; however, the priority of the right-posterior region was higher than that of the left-frontal in the SZ group. Similarly, the pinned nodes of microstate D in both the SZ and HC groups were distributed in the frontal lobe, which is consistent with the distribution in the microstate D topographic map. Surprisingly, the priority of the pinned nodes in the SZ group was significantly lower than that in the HC group. And the distribution of pinned nodes exhibited clear trends in the left and right hemispheres, with greater right-frontal than left-frontal activity in the SZ group, whereas it was distributed across the entire frontal lobe in the HC group.

## 4. Discussion

In this paper, we applied microstate, brain network and control theories to explore the network dynamics behind each microstate in first-episode schizophrenia during short-term memory tasks. Our results suggested a significant correlation between microstate and network dynamics, and the abnormal changes in brain state transitions were driven by specific microstate and brain regions in schizophrenia. We concluded that microstate D may be a biomarker candidate of abnormal brain activity during the state transitions in schizophrenia, whereas microstate B seems to act as a compensatory mechanism to maintain brain functioning and facilitate interaction with other brain regions. These findings will offer new insights into differential microstate mechanisms of cognitive control between SZ and HC groups, and provide a theoretical foundation for the application of microstates to network intervention responses in the future.

### 4.1. Abnormalities in Microstate Topology

Based on the different sensitivities of the HC and SZ groups to different microstates during the memory task, we compared the average microstate connectivity between SZ and HC groups. Obviously, each microstate connectivity distribution is consistent with the corresponding microstate topographic map, which indicates that the representative areas of each microstate have the same rich functional information exchange. Furthermore, the microstate connectivity of the SZ group tended to be stronger, which is consistent with the previous studies [35,36] that did not add the microstate. Since our network analysis is based on connectivity derived from PLV between brain regions, and phase synchrony likely reflects the coordinated activation of neural ensembles between regions, the stronger connectivity in schizophrenia might reflect an over-activation of brain regions.

In fact, many studies [37,38,39] have found that schizophrenia is related to the abnormal connectivity and activity disorders of the networks; here, we also found these abnormal dynamics by comparing the microstate topologies, suggesting a disrupted task-based mechanism in schizophrenia during memory tasks. Specifically, Cp and Eloc of the SZ group were significantly lower than those of the HC group, but they did not obey the distributions of the corresponding microstate topographic maps, and the differences were concentrated with partial right prefrontal and parietal-occipital regions in almost all microstates. We speculated that neurons in the frontal region of schizophrenia have been damaged, resulting in impaired communication between neurons. This suggests that the brain networks in the SZ group are significantly less efficient in information transmission and integration [40,41], and memory functions are abnormal, leading to their cognitive impairment. Notably, a study found that as memory load increases, the parietal and occipital lobe are activated and the power of the frontal lobe significantly increases [42]. Specifically, these significantly activated regions may have higher partial segregation, which causes the distribution of Cp and Eloc for measuring the ability of the network to transmit information at the local level [43] to overwhelm the microstate distribution that should have existed, but the global level of Lp and Eg do not have such an ability. In addition, we also found a significant correlation between microstate D and brain topologies. Overall, there are significant differences in microstate topologies between SZ and HC groups, further demonstrating a disrupted functional brain structure in schizophrenia, also supported by changes in microstate parameters, as discussed in the Supplementary Materials.

### 4.2. Abnormalities in Microstate Controllability

Our results showed that the distribution of nodes with high AC was consistent with the distribution of the corresponding microstate topographic map, whereas the high MC was inconsistent. Its consistency may reflect specific brain regions playing a key role in the transition to easy-to-reach states, whereas the inconsistency may indicate that the ability of these nodes to drive the brain to the hard-to-reach states is not entirely dependent on the corresponding microstate topologies, and may be related to compensatory network reorganization or nonlinear integration.

Specifically, the results revealed that the AC and MC of microstate D in the SZ group were significantly lower compared with those of the HC group, reflected in the frontal and occipital regions of the AC and in the right frontal and parieto-occipital lobes of the MC. This indicates that microstate D in the SZ group was significantly less capable of driving the brain to transition to both easy-to-reach and hard-to-reach states, i.e., its flexibility in processing information was reduced and its ability to perform cognitive tasks was decreased [44]. The possible reason for this is that the topologies of microstate D showed significant abnormalities in both global and local integration processes in the SZ group, resulting in a significantly reduced ability to process basic information or perform more difficult tasks. Moreover, the overall AC of microstate B was significantly lower (*p* < 0.05) but increased in the left-frontal lobe in the SZ group, suggesting a significant decrease in the overall ability of microstate B to drive the brain to easy-to-reach states but an increase in the left frontal lobe, which could be attributed to the involvement of the left frontal lobe in compensatory mechanisms [45]. However, the MC of microstate B was lower (but with no statistical difference (*p* > 0.05)), which was reflected in the right-frontal and occipital regions, suggesting a non-significant decrease in the ability of microstate B to drive the brain to hard-to-reach states. These abnormal changes in microstates not only influence the efficiency of information processing, but also aggravate cognitive impairment and symptomatic manifestations in patients, emphasizing the critical role of microstates in understanding and intervening in cognitive functioning in schizophrenia [46]. Overall, our results illustrated that the transitions in the brain’s easy-to-reach and hard-to-reach states were driven by specific microstate and brain regions.

### 4.3. Abnormalities in Microstate Synchronization

Our results suggested that microstate synchronization was significantly enhanced in the SZ group during memory tasks. We speculated that these abnormal microstate synchronizations could be indicative of brain disorders in schizophrenia, as well as a compensatory mechanism established by patients during the performance of cognitive tasks. It has been reported that microstate D is associated with the fronto-parietal network [8,9], and schizophrenia is likely related to abnormal dynamics in the prefrontal network [47,48]. Diminished activity or reduced function of the right-frontal lobe may lead to significantly enhanced connectivity among other brain regions in schizophrenia patients during memory tasks, i.e., inactive brain regions may become activated. This enhanced connectivity is likely an adaptive or compensatory mechanism to offset the lack of right-frontal lobe function [45]. Furthermore, schizophrenia is often regarded as a psychiatric disorder characterized by impaired attentional functioning. Existing evidence indicates that people’s ability to perform target detection tasks is significantly enhanced when attention is consciously directed to areas of visuospatial reasoning. Therefore, when compared to the frontal-parietal lobes, which are most prominent in memory tasks, microstate B, closely related to visual function [8,9], exhibits the highest overall synchronization phenomenon.

Importantly, we found that the frequency and coverage are significantly negatively related to synchronization in microstate D. In practice, synchronization is considered to be a global feature [29] that coordinates the entire brain network, and the microstate parameters happen to emphasize the characteristics of local regions [26]. Hence, it is not difficult to think that in the process of memory tasks, the microstate with the highest frequency and coverage happens to affect the brain’s functional networks based on this microstate to exhibit the least prominent global synchronization. This also explains why the frequency and coverage of microstate D significantly decline in patients, leading to the significant increase in their synchronization behavior. Overall, the enhanced microstate synchronization observed in the SZ group may be associated with a hyperactivation of brain function or neural abnormalities.

### 4.4. Abnormalities in Microstate Pinned Nodes

Despite significant advances in investigations of the synchronization [49] and microstate [11] of schizophrenia, the fundamental causal mechanisms remain unclear. For instance, Pan’s team [50] explored the effect of 10 Hz repetitive transcranial magnetic stimulation on the EEG microstate of schizophrenia patients, and the results revealed that the patients’ positive symptoms could be improved, and the parameters of the EEG microstate were found to be altered by stimulating the left dorsolateral prefrontal cortex. It was hypothesized that the mechanism of the improvement of the symptoms might be related to the modulation of the EEG microstate. It is worth asking whether specific microstate and brain regions are driving these changes or whether all brain regions and microstates contribute equally. Therefore, we investigated the influential microstate and brain regions that drive changes in the brain synchronization states based on synchronization pinning control to apply microstates to brain network intervention responses in future.

The distribution of the most influential nodes driving the transition of brain synchronization state is consistent with the corresponding microstate topographic maps, which indicates that the synchronization can locally characterize different microstates. Specifically, the priority of the pinned nodes in microstate B showed that the left-frontal activity was higher than that of the right-posterior in the HC group; however, the right-posterior activity was higher than the left-frontal in the SZ group. This enhanced right-lateral activity may lead to non-typical functional allocation of the brain when performing digital memory tasks, thereby interfering with normal cognitive processes. Furthermore, the distribution of pinned nodes in microstate D had obvious left and right hemisphere trends, with the right-frontal being higher than the left-frontal in the SZ group, but the pinned nodes were distributed throughout the whole frontal lobe in the HC group. This suggests a reduced level of activation in the right brain in patients, which may lead to a significant decrease in attention and memory abilities, thereby affecting cognitive function [51].

It is worth noting that the participants in this research were asked to complete a task involving memorizing numbers, which is performed with the right hemisphere [52,53]. Thus, the right hemisphere of the healthy controls was first activated during the memory task. However, the raised priority of the right hemisphere in schizophrenia patients exhibits significantly reduced synchronization compared to the left hemisphere. This finding further strengthens the hypothesis that abnormalities in the right-frontal lobe function of the patients may have triggered compensatory enhancements in other brain regions. Notably, this compensatory mechanism may have initially originated in the left hemisphere as an adaptive response to right frontal dysfunction. In addition, a study [54] observed that patients with schizophrenia have a more delayed response in processing information in the right visual field compared to the left visual field, further supporting the above view. Overall, our results demonstrated that the changes in brain synchronization state were driven by specific microstate and brain regions.

## 5. Conclusions

Previous studies have focused on isolated studies of patients’ microstate parameters or changes in network dynamics to explore the pathological mechanisms and brain dysfunctions in schizophrenia, and lacked an in-depth exploration of the relationship between network dynamics and different microstates. In addition, existing studies have not explored the particular contributions of different microstate types and specific brain regions in facilitating brain state transitions. The main contribution of this paper is to systematically explore the brain network dynamics underlying each microstate from the perspective of brain state transitions, finding that there is a significant correlation between microstates and network dynamics. Importantly, we speculated that microstate D may be a candidate biomarker of abnormal brain activity during state transitions in schizophrenia, whereas microstate B may represent a compensatory mechanism that maintains brain function and exchanges information with other brain regions. These results enhance the application potential of microstates in brain network control and provide new insights for applying microstates to mental illness interventions in the future.

There are several considerations of this study that should to be considered. First, there are four microstate types, and numbers of microstates of five or more were not considered, which may affect the comprehensiveness of the results. Second, schizophrenia is a heterogeneous condition and our relatively small sample size may not adequately cover the full schizophrenia spectrum. Inter-individual differences in EEG data may also contribute to the variability of the results, and this needs to be further investigated. Finally, in this paper, we systematically explored the relationship between microstates and network dynamics from the perspective of brain state transitions, which include easy-to-reach, hard-to-reach, and synchronized states. We will concentrate on exploring the precise brain states that are more relevant to the state of schizophrenia pathology in future studies, and on increasing the sample size and employing a variety of data collection methods to explore the relationship between microstates and network dynamics in schizophrenia in more depth, with the aim of applying microstates to the brain network response to interventions.

## Data Availability

The data that support the findings of this study are available on request from the corresponding author. The data are not publicly available due to privacy or ethical restrictions.

## Supplementary Materials

The following supporting information can be downloaded at: https://www.mdpi.com/article/10.3390/brainsci14100985/s1, Figure S1. (a) General parameters of the microstate, including average duration, amplitude, frequency, and coverage. (b) Microstate transition probability from one microstate to another for HC and SZ groups. References [55,56,57,58,59,60,61] are cited in the supplementary materials.
