# Supplementary material for: Microstate D as a Biomarker in Schizophrenia: Insights from Brain State Transitions
Source: Brain Sci. 2024 Sep 28;14(10):985. doi: 10.3390/brainsci14100985 (PMC11505886; doi:10.3390/brainsci14100985)
Supplement: Supplementary file 1 [file brainsci-14-00985-s001.zip › brainsci-3219239-supplementary.pdf]

Supplementary Materials

Microstate time series are rich in potential neurophysiological parameters. The measures include average duration, amplitude, frequency, coverage, and transition probabilities from one state to another for each microstate.

1. Analysis of Microstate Parameters

Figure S1(a) shows that microstate parameters between the healthy controls (HC) and schizophrenia patients (SZ) performed differently in different microstates. Specifically, the average duration of the four microstates showed no significant differences between the HC and SZ group, possibly because memory task is not sensitive to average duration. However, the amplitude was significantly higher in almost all microstates in SZ group. Importantly, the frequency and coverage of microstate D were lower, but these same parameters were higher in microstates A, B, and C in SZ group. For instance, microstate D was significantly lower and microstate B was significantly higher. The transition probability (horizontal → vertical) between the four types of microstates is calculated, as shown in Figure S1(b). Overall, showing that the transition probability from any microstate (A, B, C, or D) to D (HC = 0.377; SZ = 0.268) had a decreasing trend, and from any microstate (A, B, C, or D) to microstate B (HC = 0.145; SZ = 0.193) had an increasing trend in SZ group.

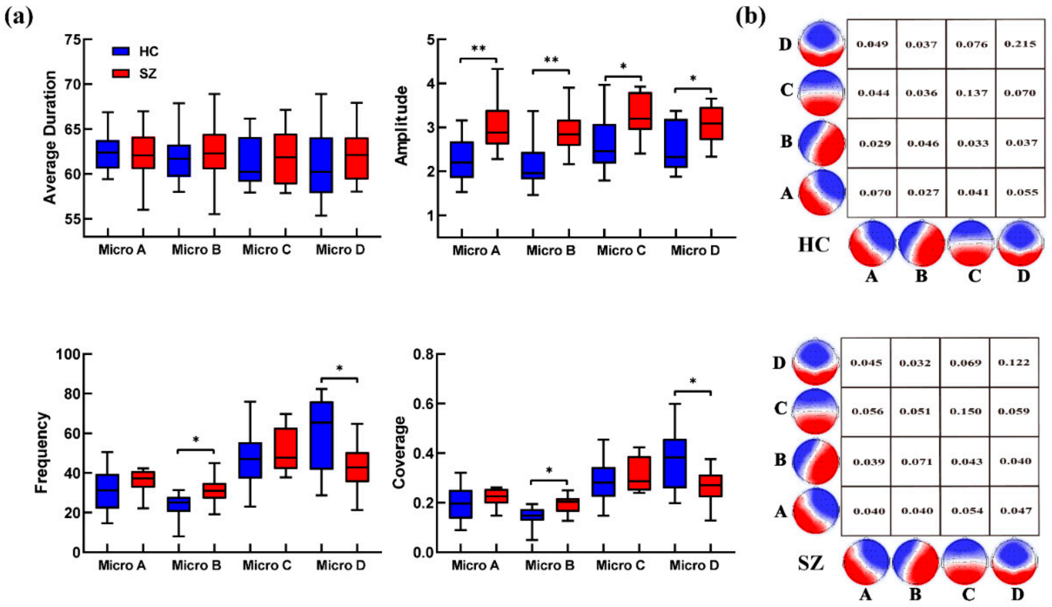

Figure S1. (a) General parameters of the microstate, including average duration, amplitude, frequency, and coverage. (b) Microstate transition probability from one microstate to another for HC and SZ groups.

2. Abnormalities in Microstate Parameters

In this paper, the frequency and coverage of microstate D were significantly reduced and microstate B were significantly increase in SZ group. The altered frequency of appearance of some microstates suggests changes in the likelihood of activation of certain neural assemblies (or changes in the activation regions that constitutes the brain network), which is verified in the transition probability. Compared with HC group, the transition probability from any microstates to microstate D was reduced, but microstate B increased in SZ group, suggesting a change in the sequential activation of underlying neural assemblies (or changes in the activation sequence that constitutes the brain network). Microstate D was associated with the fronto-parietal network (Baradits et al., 2020; Michel and Koenig, 2018), and the memory task arose in fronto-parietal circuits (Biel et al., 2022; Jun et al., 2022). Thus, reduced in the frequency and coverage of microstate D and the reduction of activation from any microstates to microstate D may indicate abnormality of the schizophrenia patients' fronto-parietal lobes, which may lead to insufficient global network operation. Furthermore, microstate B was associated with the visual network (Baradits et al., 2020; Michel and Koenig, 2018), and visual network are expected to mature much earlier than advanced cognitive network (Gogtay et al., 2004). Recent studies (Coffman et al., 2020; Tranfa et al., 2023) showed that memory task were related to the visual network, however, the frequency and coverage may not accurately characterize the rapid transition of visual function compared to memory function. Interestingly, the increase in the frequency and coverage of microstate B, along with the increased activation, as a possible sign of a hypothetically increased vulnerability, would only concern microstate B, not microstate D. This may indicate that schizophrenia patients can compensate for certain functions by increasing the frequency of microstate B. That is, in order to compensate for the reduced microstate D, increased microstate B is a compensation mechanism that maintains brain function and exchanges information with other brain regions.

In addition, there was no significant difference in the average duration of the four microstates in SZ group, whereas previous studies (Da Cruz et al., 2020; Kim et al., 2021) reported a decrease. Microstate duration is interpreted to reflect the stability of its underlying neural assemblies, and is considered as a

macro-quantitative indicator. We speculated that this may be because the duration of the microstate is less sensitive to the short-term memory task of schizophrenia; that is, first-episode schizophrenia may not be sufficient to cause a significant change in microstate duration. As the condition deteriorates, the duration of the microstate does not rule out the possibility of abnormalities. However, the amplitude (i.e., the average GFP) that reflect EEG wave frequency as an estimate of global activation showed significant enhancement in almost all microstates, indicating that the average potential values of EEG signals in SZ group were obviously higher than that of those in HC group. Notably, there was no significant difference in microstate A and C. The possible explanation is that the short-term memory task did not activate auditory network and saliency network. A study (Baradits et al., 2020) reported that A is related to the auditory network and C is related to the saliency network. Overall, microstate changes (scalp distribution variations) in schizophrenia indicated changes in the relative contribution of brain structures involved in processing memorized task. These changes seem to reflect breakdown of normal network activity, abnormal topological connectivity, decreased functional organization, or increased noise in brain processes (Lehmann et al., 2005).

3. Reference

Baradits, M., Bitter, I., Czobor, P., 2020. Multivariate patterns of EEG microstate parameters and their role in the discrimination of patients with schizophrenia from healthy controls. Psychiatry Research 288, 112938. https://doi.org/10.1016/j.psychres.2020.112938

Biel, A.L., Sterner, E., Röll, L., Sauseng, P., 2022. Modulating verbal working memory with fronto-parietal transcranial electric stimulation at theta frequency: Does it work? European Journal of Neuroscience 55, 405–425. https://doi.org/10.1111/ejn.15563

Coffman, B.A., Murphy, T.K., Haas, G., Olson, C., Cho, R., Ghuman, A.S., Salisbury, D.F., 2020. Lateralized evoked responses in parietal cortex demonstrate visual short-term memory deficits in first-episode schizophrenia. Journal of Psychiatric Research 130, 292–299. https://doi.org/10.1016/j.jpsychires.2020.07.036

Da Cruz, J.R., Favrod, O., Roinishvili, M., Chkonia, E., Brand, A., Mohr, C., Figueiredo, P., Herzog, M.H., 2020. EEG microstates are a candidate endophenotype for schizophrenia. Nat Commun 11, 3089. https://doi.org/10.1038/s41467-020-16914-1

Gogtay, N., Giedd, J.N., Lusk, L., Hayashi, K.M., Greenstein, D., Vaituzis, A.C., Nugent, T.F., Herman, D.H., Clasen, L.S., Toga, A.W., Rapoport, J.L., Thompson, P.M., 2004. Dynamic mapping of human cortical development during childhood through early adulthood. Proceedings of the National Academy of Sciences 101, 8174–8179. https://doi.org/10.1073/pnas.0402680101

Jun, S., Joo, Y., Sim, Y., Pyo, C., Ham, K., 2022. Fronto-parietal single-trial brain connectivity benefits

successful memory recognition. Translational Neuroscience 13, 506–513. https://doi.org/10.1515/tnsci-2022-0265

Kim, K., Duc, N.T., Choi, M., Lee, B., 2021. EEG microstate features for schizophrenia classification. PLoS ONE 16, e0251842. https://doi.org/10.1371/journal.pone.0251842

Lehmann, D., Faber, P.L., Galderisi, S., Herrmann, W.M., Kinoshita, T., Koukkou, M., Mucci, A., Pascual-Marqui, R.D., Saito, N., Wackermann, J., Winterer, G., Koenig, T., 2005. EEG microstate duration and syntax in acute, medication-naïve, first-episode schizophrenia: a multi-center study. Psychiatry Research: Neuroimaging 138, 141–156. https://doi.org/10.1016/j.pscychresns.2004.05.007

Michel, C.M., Koenig, T., 2018. EEG microstates as a tool for studying the temporal dynamics of whole-brain neuronal networks: A review. NeuroImage, Brain Connectivity Dynamics 180, 577–593. https://doi.org/10.1016/j.neuroimage.2017.11.062

Tranfa, M., Iasevoli, F., Cocozza, S., Ciccarelli, M., Barone, A., Brunetti, A., de Bartolomeis, A., Pontillo, G., 2023. Neural substrates of verbal memory impairment in schizophrenia: A multimodal connectomics study. Human Brain Mapping 44, 2829–2840. https://doi.org/10.1002/hbm.26248
